# 2-[(2,4-Dimethyl­phen­yl)imino­meth­yl]-6-methyl­phenol

**DOI:** 10.1107/S1600536809033637

**Published:** 2009-08-29

**Authors:** Hasan Tanak, Ferda Erşahin, Erbil Ağar, Metin Yavuz, Orhan Büyükgüngör

**Affiliations:** a Department of Physics, Faculty of Arts & Science, Ondokuz Mayıs University, TR-55139 Kurupelit-Samsun, Turkey; b Department of Chemistry, Faculty of Arts & Science, Ondokuz Mayıs University, 55139 Samsun, Turkey

## Abstract

The title compound, C_16_H_17_NO, is a Schiff base which adopts the phenol–imine tautomeric form in the solid state. The mol­ecule is almost planar, with a dihedral angle of 4.61 (4)° between the aromatic rings. The molecular structure is stabilized by an intramolecular O—H⋯N hydrogen bond which generates a six membered ring.

## Related literature

For background to the properties and uses of Schiff bases, see: Aydoğan *et al.* (2001 ▶); Barton & Ollis (1979 ▶); Layer (1963 ▶); Ingold (1969 ▶); Cohen *et al.* (1964 ▶); Moustakali-Mavridis *et al.* (1978 ▶); Taggi *et al.* (2002 ▶). For hydrogen-bond motifs, see: Bernstein *et al.* (1995 ▶). For a related structure, see: Köysal *et al.* (2007 ▶).
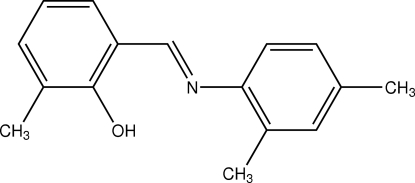


## Experimental

### 

#### Crystal data

C_16_H_17_NO
*M* *_r_* = 239.31
Monoclinic, 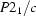

*a* = 18.1448 (11) Å
*b* = 4.7141 (3) Å
*c* = 15.7151 (8) Å
β = 99.646 (4)°
*V* = 1325.21 (13) Å^3^
*Z* = 4
Mo *K*α radiation
μ = 0.07 mm^−1^
*T* = 296 K
0.80 × 0.40 × 0.15 mm

#### Data collection

Stoe IPDS II diffractometer
Absorption correction: integration (*X-RED32*; Stoe & Cie, 2002 ▶) *T*_min_ = 0.963, *T*_max_ = 0.990
18038 measured reflections
2732 independent reflections
1808 reflections with *I* > 2σ(*I*)
*R*_int_ = 0.058

#### Refinement

*R*[*F* ^2^ > 2σ(*F* ^2^)] = 0.044
*wR*(*F* ^2^) = 0.120
*S* = 1.01
2732 reflections
169 parameters
H atoms treated by a mixture of independent and constrained refinement
Δρ_max_ = 0.10 e Å^−3^
Δρ_min_ = −0.14 e Å^−3^

### 

Data collection: *X-AREA* (Stoe & Cie, 2002 ▶); cell refinement: *X-AREA*; data reduction: *X-RED32* (Stoe & Cie, 2002 ▶); program(s) used to solve structure: *SHELXS97* (Sheldrick, 2008 ▶); program(s) used to refine structure: *SHELXL97* (Sheldrick, 2008 ▶); molecular graphics: *ORTEP-3 for Windows* (Farrugia, 1997 ▶); software used to prepare material for publication: *WinGX* (Farrugia, 1999 ▶).

## Supplementary Material

Crystal structure: contains datablocks I, global. DOI: 10.1107/S1600536809033637/fl2259sup1.cif

Structure factors: contains datablocks I. DOI: 10.1107/S1600536809033637/fl2259Isup2.hkl

Additional supplementary materials:  crystallographic information; 3D view; checkCIF report

## Figures and Tables

**Table 1:** Hydrogen-bond geometry (Å, °)

*D*—H⋯*A*	*D*—H	H⋯*A*	*D*⋯*A*	*D*—H⋯*A*
O1—H1⋯N1	0.98 (2)	1.65 (2)	2.5883 (16)	157.3 (18)

**Fig. 1.**
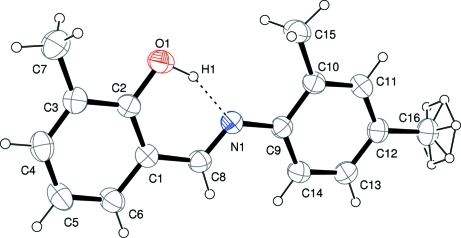
The molecular structure of the title compound, showing the atom-numbering scheme and 30% probability diplacement ellipsoids. The disordered hydrogens on C16 are also shown.

**Fig. 2.**
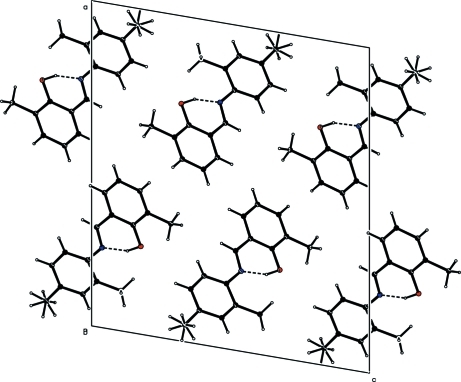
The crystal packing of the title compound. Intramolecular hydrogen bonds are shown as dashed lines.

## supplementary crystallographic information

### Comment

Schiff bases, *i.e.*, compounds having a double C=N bond, are used as starting materials in the synthesis of important drugs, such as antibiotics, antiallergic, antiphlogistic, and antitumor substances (Barton *et al.*, 1979; Layer, 1963; Ingold 1969). They also have a wide ranage of industrial uses such as dyes and pigments (Taggi *et al.*, 2002). Schiff bases have also been employed as ligands for the complexation of metal ions (Aydoğan *et al.*, 2001). Two characteristic properties of Schiff bases, are photochromism and thermochromism (Cohen *et al.*, 1964). In general, Schiff bases display two possible tautomeric forms, the phenol-imine (OH) and the keto-amine (NH) forms. Depending on the tautomers, two types of intramolecular hydrogen bonds are observed: O—H···N in the phenol-imines and N—H···O in keto-amine tautomers.

In the title compound (I, Fig. 1), the molecule is almost planar with a dihedral angle between the aromatic rings [C1/C6 and C9/C14] of 4.61 (4)°. The imino group is coplanar with the hydroxyphenyl ring as it is shown by the C2—C1—C8—N1 torsion angle of -0.9 (2)°. The O—H and C=N bond lengths confirm that (I) exhibits the enol-imine tautomer. The length of the C8=N1 double bond is 1.272 (2) Å which is slightly shorter than the standard value of 1.28 Å but it is consistent with the related sturucture (Köysal *et al.*, 2007). It is also known that Schiff bases may exhibit thermochromism depending on the planarity or non-planarity of the molecule, respectively (Moustakali-Mavridis *et al.*, 1978). Therefore, the title compound may exhibit thermochromic properties.

The phenol H atom forms a strong intramolecular O1—H1···N1 hydrogen bond with the imine N atom.  (Table 1, Fig. 1) which generates a six-membered ring, producing a S(6) ring motif (Bernstein *et al.*, 1995), stabilizing the planarity of the molecular skeleton. A packing diagram for (I) with hydrogen bonding geometry is shown in figure 2.

### Experimental

A solution of 3-methylsalicylaldehyde (0.0239 g, 0.1755 mmol) in ethanol (10 ml) was added to a solution of 2,4-dimethylaniline (0.02127 g, 0.1755 mmol) in ethanol (20 ml). The reaction mixture was stirred for 2 h under reflux. Single crystals suitable for X-ray analysis were obtained from ethyl alcohol by slow evaporation (yield 74%; m.p.386–388 K).

### Refinement

C-bound H atoms were positioned geometrically and refined using a riding model, with C—H = 0.93–0.96 Å and *U*_iso_(H) = 1.2–1.5*U*_eq_(C). The C16-methyl group was refined as idealized disordered one with two positions rotated from each other by 60°. The position of the H1 atom was obtained from a difference map of the electron density in the unit-cell and was refined freely.

### Crystal data

**Table:**

C_16_H_17_NO	*F*(000) = 512
*M**_r_* = 239.31	*D*_x_ = 1.199 Mg m^−^^3^
Monoclinic, *P*2_1_/*c*	Mo *K*α radiation, λ = 0.71073 Å
Hall symbol: -P 2ybc	Cell parameters from 21940 reflections
*a* = 18.1448 (11) Å	θ = 1.6–28.0°
*b* = 4.7141 (3) Å	µ = 0.07 mm^−^^1^
*c* = 15.7151 (8) Å	*T* = 296 K
β = 99.646 (4)°	Prism, yellow
*V* = 1325.21 (13) Å^3^	0.80 × 0.40 × 0.15 mm
*Z* = 4	

### Data collection

**Table:**

Stoe IPDS II diffractometer	2732 independent reflections
Radiation source: fine-focus sealed tube	1808 reflections with *I* > 2σ(*I*)
graphite	*R*_int_ = 0.058
Detector resolution: 6.67 pixels mm^-1^	θ_max_ = 26.5°, θ_min_ = 2.3°
rotation method scans	*h* = −22→22
Absorption correction: integration (*X-RED32*; Stoe & Cie, 2002)	*k* = −5→5
*T*_min_ = 0.963, *T*_max_ = 0.990	*l* = −19→19
18038 measured reflections	

### Refinement

**Table:**

Refinement on *F*^2^	Primary atom site location: structure-invariant direct methods
Least-squares matrix: full	Secondary atom site location: difference Fourier map
*R*[*F*^2^ > 2σ(*F*^2^)] = 0.044	Hydrogen site location: inferred from neighbouring sites
*wR*(*F*^2^) = 0.120	H atoms treated by a mixture of independent and constrained refinement
*S* = 1.01	*w* = 1/[σ^2^(*F*_o_^2^) + (0.0631*P*)^2^] where *P* = (*F*_o_^2^ + 2*F*_c_^2^)/3
2732 reflections	(Δ/σ)_max_ < 0.001
169 parameters	Δρ_max_ = 0.10 e Å^−^^3^
0 restraints	Δρ_min_ = −0.14 e Å^−^^3^

### Special details

**Table:**

Experimental. 359 frames, detector distance = 100 mm
Geometry. All e.s.d.'s (except the e.s.d. in the dihedral angle between two l.s. planes) are estimated using the full covariance matrix. The cell e.s.d.'s are taken into account individually in the estimation of e.s.d.'s in distances, angles and torsion angles; correlations between e.s.d.'s in cell parameters are only used when they are defined by crystal symmetry. An approximate (isotropic) treatment of cell e.s.d.'s is used for estimating e.s.d.'s involving l.s. planes.
Refinement. Refinement of *F*^2^ against ALL reflections. The weighted *R*-factor *wR* and goodness of fit *S* are based on *F*^2^, conventional *R*-factors *R* are based on *F*, with *F* set to zero for negative *F*^2^. The threshold expression of *F*^2^ > σ(*F*^2^) is used only for calculating *R*-factors(gt) *etc*. and is not relevant to the choice of reflections for refinement. *R*-factors based on *F*^2^ are statistically about twice as large as those based on *F*, and *R*- factors based on ALL data will be even larger.

### Fractional atomic coordinates and isotropic or equivalent isotropic displacement parameters (Å^2^)

**Table:**

	*x*	*y*	*z*	*U*_iso_*/*U*_eq_	Occ. (<1)
C1	0.35139 (7)	0.4131 (3)	0.57205 (9)	0.0555 (3)	
C2	0.33187 (7)	0.3180 (3)	0.64973 (9)	0.0568 (4)	
C3	0.37660 (8)	0.1210 (3)	0.70152 (10)	0.0639 (4)	
C4	0.43983 (8)	0.0228 (4)	0.67355 (10)	0.0716 (5)	
H4	0.4698	−0.1090	0.7072	0.086*	
C5	0.46019 (9)	0.1133 (4)	0.59720 (11)	0.0766 (5)	
H5	0.5033	0.0436	0.5800	0.092*	
C6	0.41638 (8)	0.3062 (4)	0.54709 (10)	0.0704 (4)	
H6	0.4300	0.3672	0.4956	0.084*	
C7	0.35539 (10)	0.0258 (5)	0.78536 (11)	0.0943 (6)	
H7A	0.3059	−0.0532	0.7748	0.113*	
H7B	0.3565	0.1851	0.8236	0.113*	
H7C	0.3902	−0.1155	0.8113	0.113*	
C8	0.30616 (8)	0.6172 (3)	0.51840 (9)	0.0588 (4)	
H8	0.3214	0.6770	0.4677	0.071*	
C9	0.20053 (7)	0.9169 (3)	0.48548 (8)	0.0538 (3)	
C10	0.13929 (8)	1.0247 (3)	0.51850 (9)	0.0573 (4)	
C11	0.09298 (8)	1.2164 (3)	0.46860 (9)	0.0614 (4)	
H11	0.0522	1.2882	0.4904	0.074*	
C12	0.10427 (8)	1.3069 (3)	0.38793 (9)	0.0602 (4)	
C13	0.16527 (8)	1.1959 (3)	0.35699 (10)	0.0662 (4)	
H13	0.1745	1.2516	0.3030	0.079*	
C14	0.21240 (8)	1.0049 (4)	0.40465 (9)	0.0659 (4)	
H14	0.2530	0.9335	0.3823	0.079*	
C15	0.12371 (9)	0.9339 (4)	0.60573 (10)	0.0764 (5)	
H15A	0.1653	0.9843	0.6493	0.092*	
H15B	0.1164	0.7322	0.6061	0.092*	
H15C	0.0795	1.0274	0.6174	0.092*	
C16	0.05261 (9)	1.5162 (4)	0.33607 (10)	0.0731 (4)	
H16A	0.0139	1.5690	0.3678	0.110*	0.50
H16B	0.0307	1.4313	0.2822	0.110*	0.50
H16C	0.0803	1.6818	0.3251	0.110*	0.50
H16D	0.0694	1.5524	0.2823	0.110*	0.50
H16E	0.0526	1.6901	0.3678	0.110*	0.50
H16F	0.0029	1.4395	0.3250	0.110*	0.50
N1	0.24618 (6)	0.7186 (3)	0.53786 (7)	0.0577 (3)	
O1	0.27017 (6)	0.4153 (3)	0.67706 (8)	0.0781 (4)	
H1	0.2494 (11)	0.542 (5)	0.6293 (15)	0.128 (8)*	

### Atomic displacement parameters (Å^2^)

**Table:**

	*U*^11^	*U*^22^	*U*^33^	*U*^12^	*U*^13^	*U*^23^
C1	0.0539 (7)	0.0570 (9)	0.0546 (8)	−0.0013 (6)	0.0063 (6)	−0.0050 (6)
C2	0.0524 (7)	0.0602 (9)	0.0571 (8)	−0.0024 (7)	0.0075 (6)	−0.0035 (7)
C3	0.0651 (8)	0.0648 (10)	0.0588 (8)	0.0001 (7)	0.0017 (7)	−0.0014 (7)
C4	0.0717 (9)	0.0697 (11)	0.0674 (10)	0.0120 (8)	−0.0063 (8)	−0.0044 (8)
C5	0.0661 (9)	0.0924 (13)	0.0707 (10)	0.0237 (9)	0.0096 (7)	−0.0075 (9)
C6	0.0661 (9)	0.0841 (12)	0.0623 (9)	0.0124 (8)	0.0147 (7)	0.0001 (8)
C7	0.0993 (13)	0.1095 (16)	0.0736 (12)	0.0156 (11)	0.0130 (10)	0.0261 (11)
C8	0.0604 (8)	0.0627 (10)	0.0545 (8)	0.0005 (7)	0.0127 (6)	0.0018 (7)
C9	0.0549 (7)	0.0532 (9)	0.0533 (7)	−0.0002 (6)	0.0091 (6)	−0.0012 (6)
C10	0.0584 (8)	0.0607 (9)	0.0533 (8)	−0.0004 (7)	0.0104 (6)	−0.0045 (7)
C11	0.0584 (8)	0.0647 (10)	0.0609 (8)	0.0074 (7)	0.0095 (6)	−0.0068 (7)
C12	0.0645 (8)	0.0519 (9)	0.0614 (8)	−0.0012 (7)	0.0030 (6)	−0.0043 (7)
C13	0.0730 (9)	0.0673 (10)	0.0597 (8)	0.0018 (8)	0.0153 (7)	0.0089 (8)
C14	0.0664 (9)	0.0715 (11)	0.0635 (9)	0.0094 (8)	0.0216 (7)	0.0067 (8)
C15	0.0705 (9)	0.0992 (13)	0.0635 (9)	0.0151 (9)	0.0227 (7)	0.0053 (9)
C16	0.0745 (10)	0.0645 (11)	0.0758 (10)	0.0069 (8)	−0.0003 (8)	0.0042 (8)
N1	0.0568 (7)	0.0583 (8)	0.0587 (7)	0.0037 (5)	0.0120 (5)	0.0013 (6)
O1	0.0692 (6)	0.0967 (9)	0.0732 (7)	0.0176 (6)	0.0263 (5)	0.0192 (7)

### Geometric parameters (Å, °)

**Table:**

C1—C6	1.3975 (19)	C10—C11	1.385 (2)
C1—C2	1.4005 (19)	C10—C15	1.5073 (19)
C1—C8	1.442 (2)	C11—C12	1.385 (2)
C2—O1	1.3452 (17)	C11—H11	0.9300
C2—C3	1.401 (2)	C12—C13	1.384 (2)
C3—C4	1.376 (2)	C12—C16	1.503 (2)
C3—C7	1.502 (2)	C13—C14	1.374 (2)
C4—C5	1.381 (2)	C13—H13	0.9300
C4—H4	0.9300	C14—H14	0.9300
C5—C6	1.367 (2)	C15—H15A	0.9600
C5—H5	0.9300	C15—H15B	0.9600
C6—H6	0.9300	C15—H15C	0.9600
C7—H7A	0.9600	C16—H16A	0.9600
C7—H7B	0.9600	C16—H16B	0.9600
C7—H7C	0.9600	C16—H16C	0.9600
C8—N1	1.2720 (17)	C16—H16D	0.9600
C8—H8	0.9300	C16—H16E	0.9600
C9—C14	1.3872 (18)	C16—H16F	0.9600
C9—C10	1.3985 (18)	O1—H1	0.98 (2)
C9—N1	1.4172 (17)		
			
C6—C1—C2	118.54 (13)	C13—C12—C16	121.35 (14)
C6—C1—C8	120.06 (13)	C11—C12—C16	121.60 (14)
C2—C1—C8	121.40 (12)	C14—C13—C12	121.17 (14)
O1—C2—C1	120.98 (13)	C14—C13—H13	119.4
O1—C2—C3	118.21 (13)	C12—C13—H13	119.4
C1—C2—C3	120.80 (13)	C13—C14—C9	121.15 (13)
C4—C3—C2	118.06 (14)	C13—C14—H14	119.4
C4—C3—C7	121.98 (15)	C9—C14—H14	119.4
C2—C3—C7	119.96 (14)	C10—C15—H15A	109.5
C3—C4—C5	122.17 (15)	C10—C15—H15B	109.5
C3—C4—H4	118.9	H15A—C15—H15B	109.5
C5—C4—H4	118.9	C10—C15—H15C	109.5
C6—C5—C4	119.45 (14)	H15A—C15—H15C	109.5
C6—C5—H5	120.3	H15B—C15—H15C	109.5
C4—C5—H5	120.3	C12—C16—H16A	109.5
C5—C6—C1	120.98 (15)	C12—C16—H16B	109.5
C5—C6—H6	119.5	H16A—C16—H16B	109.5
C1—C6—H6	119.5	C12—C16—H16C	109.5
C3—C7—H7A	109.5	H16A—C16—H16C	109.5
C3—C7—H7B	109.5	H16B—C16—H16C	109.5
H7A—C7—H7B	109.5	C12—C16—H16D	109.5
C3—C7—H7C	109.5	H16A—C16—H16D	141.1
H7A—C7—H7C	109.5	H16B—C16—H16D	56.3
H7B—C7—H7C	109.5	H16C—C16—H16D	56.3
N1—C8—C1	122.44 (13)	C12—C16—H16E	109.5
N1—C8—H8	118.8	H16A—C16—H16E	56.3
C1—C8—H8	118.8	H16B—C16—H16E	141.1
C14—C9—C10	119.08 (13)	H16C—C16—H16E	56.3
C14—C9—N1	124.33 (12)	H16D—C16—H16E	109.5
C10—C9—N1	116.59 (12)	C12—C16—H16F	109.5
C11—C10—C9	118.14 (13)	H16A—C16—H16F	56.3
C11—C10—C15	120.79 (13)	H16B—C16—H16F	56.3
C9—C10—C15	121.07 (13)	H16C—C16—H16F	141.1
C10—C11—C12	123.42 (13)	H16D—C16—H16F	109.5
C10—C11—H11	118.3	H16E—C16—H16F	109.5
C12—C11—H11	118.3	C8—N1—C9	123.26 (12)
C13—C12—C11	117.05 (14)	C2—O1—H1	101.3 (12)
			
C6—C1—C2—O1	179.58 (14)	C14—C9—C10—C11	−0.1 (2)
C8—C1—C2—O1	−0.3 (2)	N1—C9—C10—C11	−179.26 (13)
C6—C1—C2—C3	0.3 (2)	C14—C9—C10—C15	179.56 (15)
C8—C1—C2—C3	−179.57 (13)	N1—C9—C10—C15	0.4 (2)
O1—C2—C3—C4	−179.75 (14)	C9—C10—C11—C12	−0.1 (2)
C1—C2—C3—C4	−0.4 (2)	C15—C10—C11—C12	−179.76 (15)
O1—C2—C3—C7	−0.4 (2)	C10—C11—C12—C13	0.2 (2)
C1—C2—C3—C7	178.92 (15)	C10—C11—C12—C16	−179.85 (14)
C2—C3—C4—C5	0.4 (2)	C11—C12—C13—C14	−0.1 (2)
C7—C3—C4—C5	−178.92 (17)	C16—C12—C13—C14	179.93 (14)
C3—C4—C5—C6	−0.2 (3)	C12—C13—C14—C9	0.0 (3)
C4—C5—C6—C1	0.0 (3)	C10—C9—C14—C13	0.2 (2)
C2—C1—C6—C5	−0.1 (2)	N1—C9—C14—C13	179.27 (14)
C8—C1—C6—C5	179.76 (15)	C1—C8—N1—C9	−179.25 (13)
C6—C1—C8—N1	179.29 (14)	C14—C9—N1—C8	5.4 (2)
C2—C1—C8—N1	−0.9 (2)	C10—C9—N1—C8	−175.44 (14)

### Hydrogen-bond geometry (Å, °)

**Table:**

*D*—H···*A*	*D*—H	H···*A*	*D*···*A*	*D*—H···*A*
O1—H1···N1	0.98 (2)	1.65 (2)	2.5883 (16)	157.3 (18)
